# Enzymatic characterization of D-lactate dehydrogenase and application in alanine aminotransferase activity assay kit

**DOI:** 10.1080/21655979.2021.1972781

**Published:** 2021-09-13

**Authors:** Yi Sun, Guosheng Gao, Ting Cai

**Affiliations:** a Department of Clinical Laboratory, HwaMei Hospital, University of Chinese Academy of Sciences, Ningbo, China; b Department of Research, Ningbo Institute of Life and Health Industry, University of Chinese Academy of Sciences, Ningbo, China; c Key Laboratory of Diagnosis and Treatment of Digestive System Tumors of Zhejiang Province, Ningbo, China; d Department of Emergency, HwaMei Hospital, University of Chinese Academy of Sciences, Ningbo, China

**Keywords:** ALT kit, D-lactate dehydrogenase, stabilizer, enzymatic characterization

## Abstract

D-lactate dehydrogenase (D-LDH) is widely used for the clinical detection of alanine aminotransferase (ALT) activity. It is a key enzyme in ALT detection kits, and its enzymatic properties directly determine sensitivity and accuracy of such kits. In this study, D-lactate dehydrogenase (WP_011543503, *ld*LDH) coding sequence derived from *Lactobacillus delbrueckii* was obtained from the NCBI database by gene mining. *Ld*LDH was expressed and purified in *Escherichia coli*, and its enzyme activity, kinetic parameters, optimum temperature, and pH were characterized. Furthermore, stabilizers, including sugars, polyols, amino acids, certain salts, proteins, and polymers, were screened to improve stability of *ld*LDH during freeze-drying and storage. Finally, a kit based on *ld*LDH was tested to determine whether the enzyme had potential clinical applications. The results showed that *ld*LDH had a specific activity of 1,864 U/mg, *K_m_* value of 1.34 mM, optimal reaction temperature of 55°C, and an optimal pH between 7.0 and 7.5. When sucrose or asparagine was used as a stabilizer, freeze-dried *ld*LDH remained stable at 37°C for > 2 months without significant loss of enzymatic activity. These results indicated that *ld*LDH possesses high activity and stability. Test results using the ALT assay kit prepared with *ld*LDH were consistent with those of commercial kits, with a relative deviation <5%. These results indicated that *ld*LDH met the primary requirements for ALT assays, laying a foundation for the development of new ALT kits with potential clinical applications.

## 1. Introduction

Serum alanine aminotransferase (ALT) activity is considered a reliable and sensitive marker for the detection of liver disease [1], and also plays an important role in the prevention and monitoring of liver-related disorders such as obesity, diabetes, and cardiovascular disease [2]. ALT is found in the kidney, blood, muscle, and heart, but it is particularly abundant in liver cells, where ALT activity is 3,000 times higher than that in blood [3]. Therefore, when liver cells are injured or diseased, ALT is released, enhancing its activity in the blood. Therefore, ALT activity in the blood can be used to monitor the progression of liver disorders or other related diseases [1,4].

**Figure 1.**
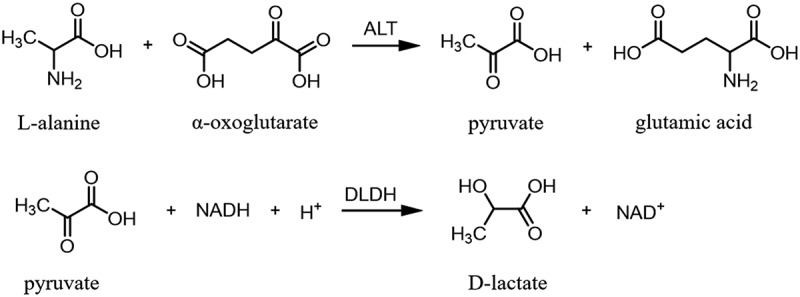
Enzyme catalysis reaction in ALT kit

To quantify ALT activity rapidly and simply, ALT activity assay kits (ALT kits) have been widely used in clinical settings. ALT kits have been extensively used to diagnose and assess liver diseases, such as nonalcoholic fatty liver disease, alcoholic liver disease, hepatitis B virus infection, drug-induced hepatotoxicity, autoimmune and cholestatic liver disease, and metabolic liver disease. It can also be used to monitor patients at risk of developing liver disease [2]. The working principle of ALT kits is shown in Figure 1. ALT in serum converts alanine to pyruvate in the first step; then, the produced pyruvate is catalyzed by lactate dehydrogenase (D-LDH) to produce lactic acid, and NADH is converted to NAD^+^. The NADH oxidation rate is proportional to ALT activity. The changed value can be monitored using a biochemical analyzer [5,6]. Therefore, only one key enzyme component, which is the activity of D-LDH, directly determines the accuracy and sensitivity of ALT kits.

However, the practical application of using D-LDH faces challenges. ALT kits are used at room temperature for clinical testing; therefore, the thermal stability of D-LDH is an important consideration for ALT kits. Additionally, purified D-LDH may lose its activity during long-term storage because of degradation or denaturation. D-LDH is derived from *Pediococcus acidilactici* [7] and *Bacillus. coagulans* [8], *Lactobacillus plantarum* [9], *L. pentosus* [10,11], *L. confusus* [12], *L. jensenii* [13], etc. However, some of the reported D-LDHs have low activity, while others have diminished thermal stability. Therefore, D-LDHs with both high activity and thermal stability are needed for clinical diagnosis.

Long-term storage stability is important for successful commercial kit application and use. Freeze-drying has been widely used to retain satisfactory bioactivity of proteins during long-term storage [14]. However, during freeze-drying, proteins suffer from stresses such as pH change, ice formation, crystal formation, and phase separation, resulting in significant changes in conformation and loss of bioactivity [14–16]. The addition of protectants is the most commonly used method for increasing bioactivity during long-term storage of proteins [17,18]. Many stabilizers, such as sugars, polyols, amino acids, certain salts, and some proteins or polymers, have been extensively studied and applied in freeze-drying and storage [14,19,20]. In a previous study, residual activity was up to 91% when organophosphorus hydrolase was stored in lyophilized form at 25°C for 60 days after adding maltose and trehalose [21] . The activity of freeze-dried catalase recovered in the presence of sucrose was up to 96.3%, higher than that in the presence of glycerol, sorbitol, and dextran [14]. However, the protective effects of these stabilizers vary from protein to protein depending on other parameters, such as storage pH, temperature, and concentration of additives [14]. Therefore, the optimal protectant for a protein needs to be screened for freeze-drying and storage.

To obtain a novel D-LDH with high specific activity and elevated thermal stability, a series of D-LDHs from different species indexed in the NCBI database were mined and screened. Only one D-LDH was selected per species. We heterologously expressed these dehydrogenases in *Escherichia coli* (*E. coli*). The yield and activity of these enzymes were evaluated (data not shown). Finally, we selected D-LDH derived from *L. delbrueckii* (*ld*LDH) for further systematic studies. *Ld*LDH (WP_011543503) was expressed and purified in *E. coli*, and its enzymatic properties were characterized. To further improve storage stability, freeze-drying was performed. A total of 22 stabilizers belonging to the six categories mentioned above were screened during freeze-drying and storage. Finally, ALT kits based on *ld*LDH were prepared according to commercial standards and compared with commercial kits. *Ld*LDH laid the foundation for the development of a new ALT kit with potential clinical applications.

## 2. Materials and methods

### 2.1. Strains and plasmids

*E. coli* BL21 (DE3) cells (Novagen, Madison, WI, USA; genotype: F ^–^ ompT hsdS_B_ (r_B_^−^m_B_^−^) gal dcm(DE3); GenBank accession No. CP001509) were used as hosts for protein expression. The amino acid sequence of D-lactate dehydrogenase (WP_011543503, *ld*LDH) of *L. delbrueckii* was obtained from the NCBI database. The full-length gene, containing an *Nco*I restriction site at the 5ʹ-end and an *Xho*I restriction site at the 3ʹ-end, was obtained by total gene synthesis (Genewiz, Suzhou, China). The synthetic gene was digested with *Nco*I and *Xho*I and inserted into *Nco*I/*Xho*I-digested pET-28a(+) plasmid, and the construct was confirmed by DNA sequencing (Genewiz) and referred to as pET-28a-*ld*LDH. The C-terminus of the *ld*LDH protein contained a His_6_-tag, which allows for convenient purification using Ni^2+^-NTA columns.

### 2.2. *Expression and purification of* ld*LDH*

*Ld*LDH was expressed and purified as described by Zhou et al. [22], with certain modifications. The pET-28a-*ld*LDH was transformed into *E. coli* BL21 (DE3) using the heat shock method, and a recombinant *ld*LDH-expressing strain was constructed. A single colony of the *ld*LDH recombinant strain was randomly selected and placed into 5 mL of LB medium (10 g/L peptone, 5 g/L yeast powder, and 10 g/L NaCl) with 50 μg/mL kanamycin. After overnight incubation at 37°C, the seed culture was inoculated into 100 mL of LB medium with 1% inoculum. Further incubation at 37°C was performed until the cell density reached 0.6–0.8 (OD_600_). The expression conditions were optimized at different induction temperatures (16°C, 25°C, and 37°C) and IPTG concentrations (0.1 mM, 0.5 mM, and 1 mM). Then, recombinant *ld*LDH was expressed by adding IPTG to a final concentration of 0.1 mM and incubation at 25°C for 12 h using 1 L LB medium.

Bacteria were collected by centrifugation at 5,500 × g resuspended in lysis buffer (50 mM Tris/HCl, 100 mM NaCl, 20 mM imidazole, pH 8.0), and homogenized using a high-pressure homogenizer (ATS, Taizhou, China). The solution was centrifuged at 26,000 × g at 4°C for 30 min to remove insoluble precipitates. The supernatant was purified using a nickel affinity chromatography column (Ni^2+^-NTA, GE, USA). Washing was performed using five column volumes of washing buffer (50 mM Tris/HCl, 100 mM NaCl, 40 mM imidazole, pH 8.0), followed by elution buffer (50 mM Tris/HCl, 100 mM NaCl, 200 mM imidazole, pH 8.0) to elute the *ld*LDH target protein. After elution, the target protein was placed in a dialysis tube and dialyzed against dialysis buffer (50 mM Tris/HCl, 100 mM NaCl, pH 8.0). The final protein purity was detected using 12% SDS-PAGE (Epizyme, Shanghai, China), and the protein concentration was determined using the BCA protein quantification kit (CWBIO, Beijing, China), which utilizes Cu^2+^, which can be reduced to Cu^+^ by proteins under alkaline conditions. Cu^+^ interacts with bicinchoninic acid (BCA), producing a sensitive color reaction and forming a purple complex, which can be monitored optically at 562 nm [23].

### 2.3. *Enzymatic characterization of* ld*LDH*

#### 2.3.1. Reaction system

The absorbance of the *ld*LDH cofactor NADH at 340 nm was used to quantify the reaction rate [24] and characterize *ld*LDH activity. The *ld*LDH enzyme activity reaction system was modified as previously described [9,25]. Briefly, the reaction system consisted of 10 mM pyruvate, 0.02 mM NADH, and 50 mM Tris/HCl (pH 7.5), and 0.05 U *ld*LDH was added to initiate the reaction. The absorbance of the reaction system was measured at 340 nm using a spectrometer (SpectraMax M5, CA, USA) over 5 min in a total volume of 200 μL. Enzymatic activity was defined as the amount of enzyme required to oxidize 1 μmol of NADH per minute [26]. The total enzyme activity and specific enzyme activity of the reaction system were calculated using an NADH extinction coefficient of 6.22 cm^2^/μmol [5,26]. One unit of activity was defined as the amount of enzyme that catalyzed the oxidation of 1 μmol of NADH per minute under standard conditions (37°C, pH 7.5).

#### 2.3.2. *Optimum temperature and pH of* ld*LDH*

Using the above reaction system, enzyme activity was measured at different reaction temperatures to determine the optimal reaction temperature [27]. The temperature was set to 25°C, 35°C, 40°C, 45°C, 50°C, 55°C, 60°C, and 70°C, while the pH was set to 7.5, and all other conditions remained unchanged. The change in absorbance at 340 nm was monitored every 10 s using a microplate analyzer. To determine the optimal pH, the above reaction system was set at 37°C, and changes in enzyme activity were determined over a series of different pH values (5.0–11.0). Changes in absorbance at 340 nm were monitored every 10 s using a microplate analyzer. The different pH buffers used were set as follows: 100 mM citrate buffer (pH 5.0–6.0), 100 mM phosphate buffer (pH 6.0–7.5), 100 mM Tris buffer (pH 7.5–9.0), and 100 mM carbonate buffer (pH 10.0–11.0). Each experiment was repeated three times.

The effects of different metal ions (Na^2+^, K^+^, Ca^2+^, Fe^3+^, Mn^2+^, Mg^2+^, Zn^2+^, Ba^2+^, Hg^2+^, Cu^2+,^ and Co^2+^) and other additives (ethanol and EDTA) on the enzymatic activity of *ld*LDH were investigated. *Ld*LDH (100 μg) was mixed with additives (1 mM, 1% for ethanol) at 30 C for 1 h, and activity was measured as described above. Enzyme activity in the absence of any additives was used as the control (100%).

### 2.4. *Determination of* ld*LDH kinetic parameters*

The determination of *ld*LDH kinetic parameters was performed according to a previous study [7], with some modifications. At 37°C, pH 7.5, different concentrations of pyruvate (0.0625 mM, 0.125 mM, 0.25 mM, 0.5 mM, 1.0 mM, 2.0 mM, 4.0 mM, 8.0 mM, 10.0 mM, 20.0 mM) were added into the enzyme activity reaction system. Changes in absorbance at 340 nm were monitored using a microplate analyzer. The kinetic parameters (*K_m_* and *V_max_*) were calculated using the Lineweaver-Burk (double reciprocal) plot using GraphPad Prism 6.01 (GraphPad Software, CA, USA).

### 2.5. *Determination of* ld*LDH stability and stabilizer selection*

#### 2.5.1. *Stability of* ld*LDH*

*Ld*LDH stability was determined based on its pH tolerance and thermal stability [22]. For pH tolerance, the purified enzyme was concentrated and placed in a buffer solution at a pH of 3–11 by ultrafiltration. The enzyme solution was incubated at 4°C for 30 min, the precipitate was removed by centrifugation, and a certain volume of the supernatant was collected to determine enzyme activity, as previously described. To determine thermal stability, the purified enzyme was placed in a water bath at different temperatures, allowed to stand for 20 min, and a certain volume of supernatant was taken to determine enzyme activity, as previously described. Each experiment was repeated three times.

#### 2.5.2. *Screening of* ld*LDH stabilizers*

A total of 22 stabilizers were identified from the literature and screened for improved stability of *ld*LDH after freeze-drying [19,20]. These protective agents can be divided into six groups: (i) sugars, including sucrose, trehalose, glucose, lactose, and sorbose; (ii) amino acids, including glycine, lysine, asparagine, methionine, threonine, and glutamate; (iii) polyols, including sorbitol and xylitol; (iv) salts, including potassium gluconate, EDTA, NaCl, KCl, MgCl_2_, CaCl_2_, and citric acid; (v) protein, bovine serum albumin (BSA); and (vi) polymer, polyethylene glycol (PEG-2000). A specific concentration of each stabilizer was mixed with 5 mg/mL *ld*LDH solution and frozen overnight at −80°C. The sample was vacuum-dried at −40°C using a freeze-dryer (Labconco Freezone 18 L, Kansas, USA) for 48 h. The lyophilized samples were treated and dissolved in 100 mM phosphate buffer (pH 7.5) and used to determine enzyme activity. Some of the samples were placed in an incubator at 37°C for accelerated temperature testing, while the remaining samples were preserved for use in measuring enzyme activity after 60 days.

### 2.6. *Preparation of ALT kit using* ld*LDH*

*Ld*LDH was mixed with optimal stabilizer and freeze-dried at −40°C. The performance of *ld*LDH was tested using a blank ALT kit (Mindray, Shenzhen, China) without lactate dehydrogenase, including Tris buffer, L-alanine, NADH, α-ketoglutarate, and preservatives [28]. After adding the prepared 20 kU/L *ld*LDH in a blank ALT kit, a biochemical analyzer (Siemens, ADVIA2400, München, Germany) was used to determine fresh human serum samples. A commercial ALT test kit (Mindray, Shenzhen, China) was used as a positive control to determine serum samples. A total of 80 human serum samples from 64 (81.25%) healthy individuals and 15 (18.75%) hepatopathy patients were included in this study, including 3 (3.75%) patients with severe hepatic disease exhibiting high ALT activity (> 200 U/L). All patients were admitted to Hwa Mei Hospital, University of Chinese Academy of Science (Ningbo, China) between April and May 2021.

### 2.7. Statistical analysis

All experimental data in this study are the average values of measurements taken in triplicate. Analysis of variance (ANOVA) was carried out using GraphPad Prism 6.01 (GraphPad Software) followed by Dunnett’s multiple comparison analysis. Statistical significance was set at p < 0.05. The kinetic parameters (*K_m_* and *K_cat_*) were calculated using Lineweaver-Burk plots.

## 3. Results

In this study, a new ALT kit was developed based on a novel *ld*LDH derived from *L. delbrueckii*. The enzymatic properties and thermostability of *ld*LDH, expressed and purified in *E. coli*, were systematically characterized. To further improve its application prospects, stabilizers were screened for preservation of activity during freeze-drying and long-term storage. Finally, the ALT kit was prepared and used to detect ALT activity in serum.

### 3.1. *Expression and purification of* ld*LDH*

**Figure 2.**
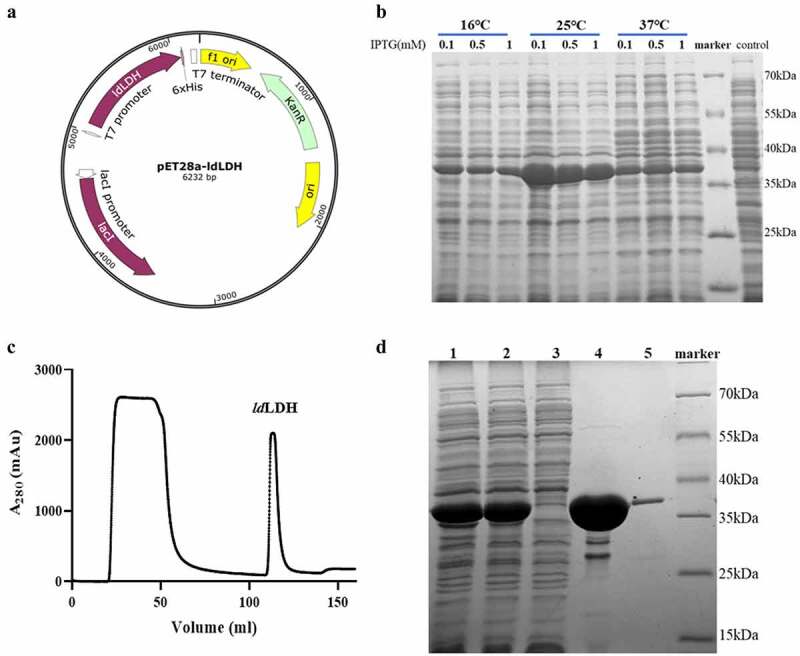
Expression of *ld*LDH in *E. coli* and purification analysis

The synthesized *ld*LDH gene fragment was inserted into pET-28a(+) to form the pET-28a-*ld*LDH expression plasmid. A His_6_-tag was included at the C-terminus of *ld*LDH to minimize any effects on *ld*LDH activity (Figure 2(a)). The pET-28a-*ld*LDH was transformed into *E. coli* BL21 (DE3) cells, and the incubation temperature and concentration of IPTG were optimized for maximal recombinant protein expression. The results showed that the highest level of *ld*LDH was expressed and presented in the supernatant in soluble form when induction was performed using 0.1 mM IPTG at 25°C (Figure 2(b)). LB medium was used to express high amounts of *ld*LDH based on previously described optimized expression conditions. A pure form of *ld*LDH was obtained following purification of the homogenized supernatant by Ni^2+^-NTA affinity column isolation due to the presence of a His_6_-tag at the C-terminus of *ld*LDH (Figure 2(c)). As shown in the chromatogram, the first peak was the flow-through fraction, which did not bind to the Ni^2+^-NTA column. The second peak represented eluted *ld*LDH. SDS-PAGE analysis showed that the molecular weight of *ld*LDH was 30 kDa, which is consistent with the predicted molecular weight (Figure 2(d)). Pure *ld*LDH was subsequently used for analysis of enzymatic properties, stabilizer screening, and ALT kit development.

a) Schematic description of pET-28a-*ld*LDH expression plasmid. b) Expression of *ld*LDH at different temperatures and induction concentrations: the temperature was set at 16°C, 25°C, and 30°C; IPTG was used for expression induction at three different concentrations, 0.1 mM, 0.5 mM, and 1.0 mM. A strain cultured at 25°C without IPTG was used as control. After overnight incubation, the supernatant was used for SDS-PAGE analysis. c) Purification of *ld*LDH with a C-terminal His_6_-tag by nickel affinity chromatography. D) SDS-PAGE analysis of purified *ld*LDH. lane 1, crude extract; lane 2, supernatant; lane 3, flow through; lane 4–5, purified *ld*LDH.

### 3.2. *Optimum temperature and pH of* ld*LDH*

**Figure 3.**
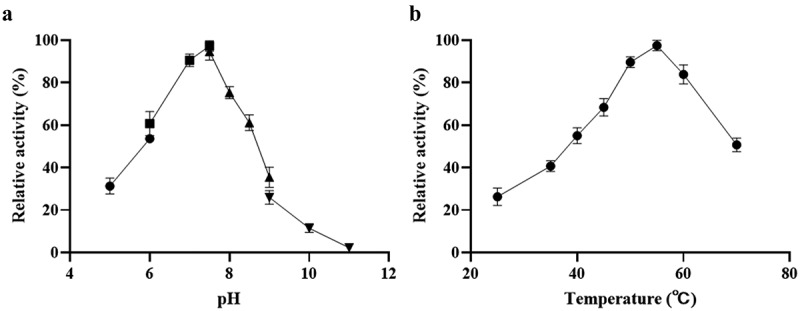
pH and temperature dependence of *ld*LDH activity

*Ld*LDH catalyzes the conversion of pyruvate to lactic acid, and the cofactor NADH is oxidized to NAD^+^. NADH in solution has a strong absorbance peak at 340 nm, and can thus be used to monitor changes in NADH concentration. Therefore, the catalytic activity of *ld*LDH can be determined based on levels of reduced NADH. The reaction system was incubated at different temperatures to determine the influence of ambient temperature on *ld*LDH activity (Figure 3(b)). The highest activity was observed at 55°C, and further increases in temperature led to a decline in enzymatic activity. Similarly, the highest *ld*LDH activity was observed at pH 7.0–7.5, and subsequent increases in pH led to a rapid decrease in enzyme activity (Figure 3(a)). These results also showed that phosphoric acid buffer or Tris buffer did not have any significant effect on enzyme activity. As a result, the enzyme remained stable at pH 7.5, which met the kit requirements.

a) Effect of pH on the activity of *ld*LDH. Citrate buffer (pH 5.0–6.0), phosphate buffer (pH 6.0–7.5), Tris buffer (pH 7.5–9.0), carbonate buffer (pH 10.0–11.0). b) Effect of temperature on *ld*LDH activity. The activity of *ld*LDH was determined at the indicated temperatures. Data are expressed as a percentage of enzyme activity as assayed at 55°C. Error bars in the Figure indicate standard deviation from three parallel replicates.

### 3.3. *Effects of additives on* ld*LDH activity*

The effects of metal ions and other additives on the activity of *ld*LDH were characterized. Residual activity was assayed following incubation with metal ions, EDTA, or ethanol (Table 1). Mg^2+^ and Mn^2+^ slightly stimulated *ld*LDH activity. To some extent, Ca^2+^, Fe^3+^, Zn^2+^, Ba^2+^, and ethanol inhibited enzymatic activity. Hg^2+^ and Cu^2+^ significantly inhibited *ld*LDH activity. The other additives had no obvious effects on *ld*LDH activity. Taken together, *ld*LDH is a metal ion-independent enzyme, because EDTA chelates metal ions and had no significant effect on *ld*LDH activity.

**Table 1.** Effects of additives on *ld*LDH activity

No.	Additive	Conc.	Relative activity (%)
1	-	0	100.0 ± 0.6
2	NaCl_2_	1 mM	99.1 ± 1.6
3	KCl	1 mM	99.5 ± 1.8
4	CaCl_2_	1 mM	91.6 ± 2.4
5	MnCl_2_	1 mM	106.6 ± 5.1
6	FeCl_3_	1 mM	86.7 ± 4.4
7	MgCl_2_	1 mM	102.5 ± 3.1
8	ZnCl_2_	1 mM	96.8 ± 3.7
9	BaCl_2_	1 mM	82.5 ± 2.9
10	HgCl_2_	1 mM	43.8 ± 2.7
11	CuCl_2_	1 mM	63.2 ± 3.5
12	CoCl_2_	1 mM	97.5 ± 2.8
13	NiCl_2_	1 mM	96.9 ± 3.8
13	EDTA	1 mM	98.6 ± 5.2
14	Ethanol	1%	86.3 ± 3.3

### 3.4. *Kinetic characterization of* ld*LDH*

**Figure 4.**
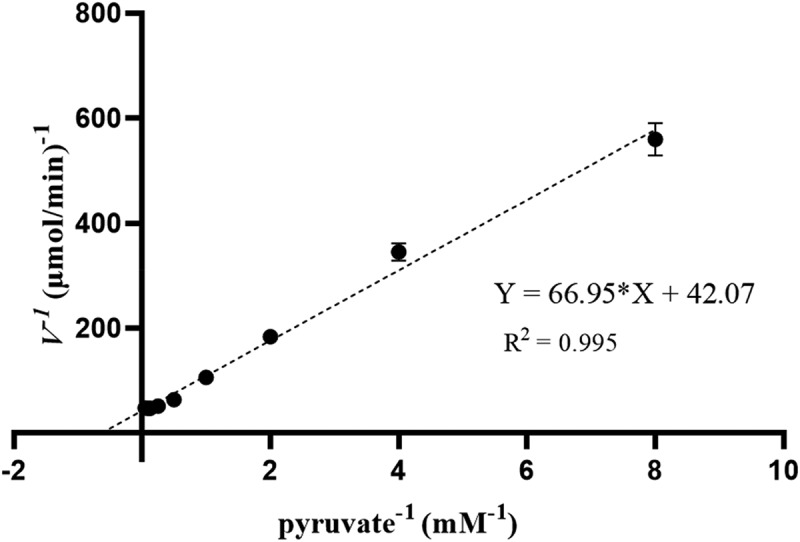
Lineweaver-Burk plot of *ld*LDH

The catalytic reaction rates of *ld*LDH at different concentrations of pyruvate were determined using the reaction system described above. When the pyruvate concentration was 10 mM, the reaction rate reached its maximum, and *V_max_* was 0.024 μmol/min. The specific enzyme activity of *ld*LDH was 1,864 U/mg at 10 mM pyruvate and 37°C, which was significantly higher than that reported previously (Table 2). The *K_m_* value of *ld*LDH for pyruvate was 1.34 mM, the *K_cat_* value was 1,603 s^−1^, and the *K_cat_*/*K_m_* value was 1,198 mM^−1^ ·s^−1^, which were calculated using the Lineweaver-Burk method (Figure 4).

**Table 2.** Comparison of biochemical properties of D-LDH from various strains

Strain	Specific activity (U/mg)	Optimum temperature (°C)	Optimum pH	*K_m_* (mM)	*K_cat_* (s^−1^)	*K_cat_*/*K_m_* (mM^−1^ s^−1^)	Reference
*Lactobacillus delbrueckii*	1864	55	7.0–7.5	1.34	1603	1198	This study
*Pediococcus acidilactici*	442	30	5.5	0.09	287	3157	[7]
*Pediococcus pentosaceus*	835	45	5.5	0.49	320	658	[37]
*Lactobacillus. coryniformis*	1206	ND^a^	ND	ND	ND	ND	[27]
*sporolactobacillus inulinus*	81.8	ND	5.5	ND	ND	ND	[36]
*Lactobacillus. pentosus*	18.6^b^	ND	ND	0.12	321	2675	[10,11]
*Lactobacillus. confusus*	ND	45	6.0	0.68	ND	ND	[37]
*Bacillus. coagulans*	ND	ND	ND	2.2	23.6	11	[8]

^a^ND: not detected.

^b^The value was detected using crude enzyme.

**Table 3.** *Ld*LDH stabilizer screening

No.	Stabilizer	Concentration	Relative activity after Lyophilization (%)	Relative activity after preserved at 37°C for 60 days (%)
1	-	0	100.0 ± 0.5	47.3 ± 2.9
2	Sucrose	5%	100.7 ± 3.2	99.5 ± 3.4
3	Trehalose	5%	100.2 ± 2.7	85.3 ± 4.1
4	Glucose	5%	99.4 ± 4.6	16.6 ± 1.1
5	Lactose	5%	94.3 ± 3.8	21.8 ± 0.7
6	Potassium gluconate	5%	101.6 ± 5.0	81.9 ± 4.6
7	Sorbose	150 mM	87.1 ± 3.6	5.7 ± 0.2
8	Sorbitol	200 mM	116.2 ± 6.2	88.9 ± 3.7
9	Xylitol	200 mM	121.0 ± 6.6	87.6 ± 5.1
10	Glycine	100 mM	84.1 ± 3.9	82.7 ± 4.8
11	Lysine	100 mM	84.7 ± 3.1	3.8 ± 0.1
12	Asparagine	100 mM	96.0 ± 4.2	99.1 ± 3.7
13	Methionine	100 mM	87.8 ± 3.2	77.8 ± 4.2
14	Threonine	100 mM	91.8 ± 5.1	74.4 ± 3.9
15	EDTA	1 mM	109.0 ± 6.3	84.2 ± 4.6
16	NaCl	50 mM	94.2 ± 5.6	79.2 ± 3.2
17	KCl	50 mM	102.1 ± 4.9	76.0 ± 3.9
18	MgCl_2_	50 mM	83.1 ± 3.9	14.3 ± 1.2
19	CaCl_2_	50 mM	80.6 ± 5.2	88.2 ± 5.7
20	BSA	1%	108.6 ± 6.8	70.3 ± 5.1
21	PEG-2000	5%	80.7 ± 4.7	64.9 ± 3.9
22	Glutamate	100 mM	111.2 ± 6.3	79.7 ± 4.1
23	Citric acid	200 mM	101.8 ± 5.2	87.3 ± 3.5

### 3.5. *Stability determination of* ld*LDH*

**Figure 5.**
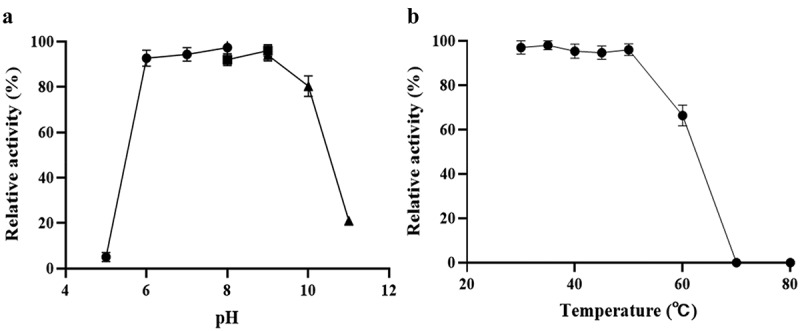
Effects of pH and temperature on *ld*LDH stability

*Ld*LDH was incubated at different temperatures and pH conditions for specific periods, and residual activity was measured to determine thermal stability and pH tolerance. As shown in Figure 5(a), *ld*LDH was stable between pH 5.5 and 9.0, and 90% of enzyme activity was retained. However, at pH < 5, activity was lost, while at pH 10, > 80% of the catalytic activity was retained, and at pH 11, only 20% of the activity was retained. As shown in Figure 5(b), when *ld*LDH was placed below 50°C, the enzyme activity was stable, and no loss was observed. However, an increase in temperature led to a sharp decrease in enzyme activity, and enzyme activity was lost at 70°C. However, *ld*LDH retained more than 60% of its activity at 60°C. Its thermal stability was found to be better than that of lactate dehydrogenase from other strains, such as *L. janssenii* [13] and *L. plantarum*.

a) Stability of *ld*LDH was determined by incubating for 30 min at the indicated pH value and then assayed at pH 8.0. Data are expressed as a percentage of maximal enzyme activity. b) Effect of temperature on the stability of *ld*LDH. Error bars in the figure indicate standard deviation of three parallel replicates.

### 3.6. *Selection of an optimal stabilizer for* ld*LDH*

Freeze-drying enzymes is beneficial for preservation and transportation, but sometimes leads to loss of enzyme activity [15], and the addition of a protective agent is needed for protein stabilization [29,30]. A total of 22 stabilizers, including sugars, polyols, amino acids, salts, proteins, and polymers, were identified from literature sources and used in the experiments reported here (Table 3) [31,32]. The stabilizers were each mixed with *ld*LDH in specific proportions and freeze-dried. Freeze-dried samples were used to determine residual enzyme activity. After freeze-drying, 12 of the 22 stabilizers were found to significantly protect *ld*LDH by retaining > 95% of *ld*LDH activity. Sorbitol, xylitol, and glutamate effectively reduced the loss of activity caused by lyophilization and significantly increased *ld*LDH activity by 10%–20% compared to the group without a stabilizer (p < 0.05). For accelerated high-temperature testing, the lyophilized samples were subjected to an ambient temperature of 37°C to determine the stability of *ld*LDH. Ten of the 22 protective agents were found to be effective in protecting *ld*LDH for 60 days and retained enzyme activity by > 80%. Notably, when sucrose or asparagine was used as a stabilizer, there was almost no loss of *ld*LDH activity after 60 days (p > 0.68). In contrast, when lysine, sorbose, and magnesium chloride were used as stabilizers, *ld*LDH activity was significantly reduced (p < 0.05). Therefore, to retain maximal *ld*LDH activity, sucrose and asparagine are considered the preferred stabilizers.

### 3.7. *Performance of* ld*LDH in ALT Kit*

**Figure 6.**
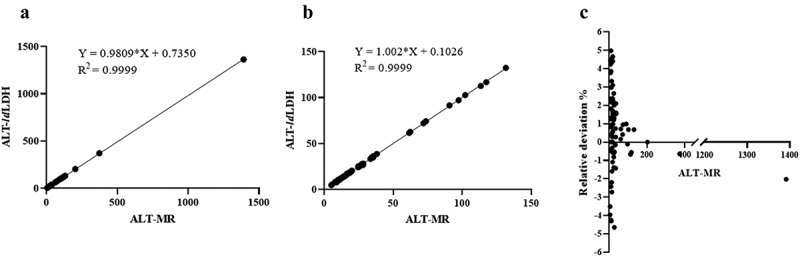
Performance of *ld*LDH in ALT kit

To verify the potential clinical application of *ld*LDH, with sucrose employed as a stabilizer, the freeze-dried powder was added to an ALT-blank kit (without *ld*LDH), and serum ALT activity was measured using a biochemical analyzer. At the same time, a commercial ALT kit (ALT-MR) was used to determine serum ALT activity and a comparison of the accuracy of the *ld*LDH-based ALT kit (ALT-*ld*LDH) was conducted. As shown in Figures 6a and 6b, the serum ALT activity detected using the developed ALT kit exhibited a highly linear relationship, with values detected using the commercial Mindray ALT kit, with an R^2^ value of 0.9999. Analysis of the relative deviation degree of the two kits revealed that the relative deviation degree was <5%, which met the requirements of the ALT kit (Figure 6(c)). Furthermore, outliers with ALT activity >200 can be accurately detected, with a relative deviation <3%, including 1,391 in one group (Figures 6a and 6c). Therefore, the developed ALT kit can be used to detect the activity of serum ALT with high sensitivity and accuracy.

Serum ALT activity from 3–1,400 U/L (a) and 3 − 150 U/L (b) was measured using the ALT kit with pure *ld*LDH (ALT-*ld*LDH) and commercial ALT kit (ALT-MR). **C**) The relative deviation between ALT-*ld*LDH and ALT-MR was determined using a commercial kit (ALT-MR) as a standard control.

## 4. Discussion

ALT is an important diagnostic indicator, and it provides a simple and reliable method for monitoring liver function during acute liver injury circumstances such as viral hepatitis and toxic hepatitis [33,34]. D-LDH is the core enzyme involved in the preparation of ALT kits, and its activity and stability affect the performance of such kits. However, the D-LDH reported in previous studies has low activity or stability, limiting its practical application. To the best of our knowledge, there are few studies regarding the systematic investigation of D-LDH biochemical properties. Here, a novel *ld*LDH derived from *L. delbrueckii*w was cloned, expressed, purified, and systematically characterized.

The *ld*LDH in this study showed excellent enzymatic properties and high thermostability. The specific activity of *ld*LDH was 1,864 U/mg, which was significantly higher than that of D-LDHs from other strains reported previously (Table 1). *Ld*LDH has moderate kinetic parameters, which are lower than those of several other D-LDHs, indicating that there is a considerable improvement space by site-directed mutagenesis. Therefore, the bioactivity and kinetic parameters of *ld*LDH can be further improved by directed evolution methods, such as error-prone PCR, rational design, and DNA shuffling, based on sequence alignment, homology modeling, and computational analysis [35]. The *K_m_* value of D-LDH from *Sporolactobacillus inulinus* was reduced from 0.114 mM to 0.08 mM by an N174Y mutation designed based on crystal structure analysis [36].

In addition, *ld*LDH exhibited high thermostability. When *ld*LDH was incubated at 50°C for 20 min, *ld*LDH retained most of its catalytic activity, while at 60°C, > 60% of its activity was retained. D-LDH derived from *Pediococcus acidilactici* is reported to exhibit a rapid decrease in enzyme activity at temperatures > 30°C [7]. The T_50_ value (the temperature at which 50% of enzyme activity is lost following heat treatment for 10 min) of D-LDH derived from *L. coryniformis* is 39.5°C [27]. The activity of D-LDH derived from *P. pentosaceus* is markedly decreased at 45°C [37]. The D-LDH identified here by genomic mining was stable at 70°C with a half-life of 84 h, however, its specific activity was only 30.2 U/mg [38], much lower than that of *ld*LDH. Therefore, to meet the requirements for clinical testing, both high thermostability and activity are required, and the developed *ld*LDH meets the requirements for clinical diagnosis.

To improve the chemical and physical stability of proteins that facilitate commercial distribution and storage, the freeze-drying method has been widely used. Additionally, after the freeze-drying of proteins, the costs involved in product preservation and transportation can be reduced. However, during freeze-drying, protein structural perturbation, aggregation, denaturation, or loss of activity may be observed due to a variety of stresses such as crystallization, dehydration stress, interface stress, pH change, ionic strength change, and ice crystal formation [39,40]. Therefore, it is necessary to add protective agents to stabilize the protein, protect the protein native structure from denaturation, and reduce the loss of enzyme activity [29]. Various compounds, including sugars, polyols, amino acids, certain salts, proteins, and polymers have been proven to be effective in minimizing protein denaturation during the freeze-drying process [20,21,40].

To date, several theories have been proposed to explain the protective mechanisms responsible for the effects of protectants on proteins during lyophilization. Vitrification (glass formation) and water replacement theory are the two main mechanisms [40]. The vitrification mechanism depends on the immobilization of protein molecules, accompanied by glassification, preventing protein-protein interactions. The latter mechanism involves the formation of hydrogen bonds between stabilizers and polar groups of protein molecules, inhibiting the unfolding of proteins. Other stabilization mechanisms include ligand binding, protectant-protein interactions via amino protons, formation of hydration spheres, and accumulation of stabilizers around specific amino acid types [14,40–42].

In this study, the effects of different stabilizers on the freeze-drying and long-term storage stability of *ld*LDH were tested. During freeze-drying, sorbitol, xylitol, and glutamate exhibited good protective effects against *ld*LDH denaturation. However, when *ld*LDH was stored at 37°C, the bioactivity of freeze-dried *ld*LDH mixed with sorbitol, xylitol, and glutamate were significantly reduced. In contrast, sucrose and asparagine maximally preserved the bioactivity of freeze-dried *ld*LDH stored at 37°C for 2 months. This difference may be attributed to several factors, such as the glass transition temperature (Tg), stabilizer phase separation, and additional stress factors during storage. The Tg of the stabilizer is directly related to its long-term stabilizing ability; sucrose may have a higher Tg than sorbitol, xylitol, and glutamate. High Tg may contribute to maintaining the freeze-dried *ld*LDH in a stable, glassified state during storage [43,44]. Additionally, the observed decline in protective action may result from a greater tendency to undergo phase separation compared with sucrose and asparagine [45,46]. Furthermore, additional stress factors during storage, such as unintended temperature excursions, may result in different protective effects between freeze-drying and long-term storage [46].

In terms of practical applications, the solubility of asparagine is relatively low and may cause a small amount of precipitation at different environmental temperatures. Moreover, the addition of asparagine can lead to a significant change in pH, which needs to be readjusted. Therefore, we selected sucrose, which is more stable and convenient for further study. Notably, sucrose preserved 99.5% of *ld*LDH activity after 60 days of storage. To the best of our knowledge, this is the most stable *ld*LDH reported to date. Our findings were in accord with previous studies in which a significant protective effect of sucrose was observed on protein stability during freeze-drying and storage [14,40,47]. The mechanism by which sucrose enhances the stability of *ld*LDH may involve remaining amorphous, water replacement, and vitrification [43,44], as described above. *Ld*LDH with high stability has great advantages in clinical applications. This is because when ALT kits are used for liver function tests, they are generally performed at room temperature, and *ld*LDH, which is highly stable, can help obtain reliable data, which is highly significant in clinical detection of disease.

Finally, freeze-dried *ld*LDH was used to prepare an ALT assay kit, and its clinical application was compared with that of a commercial kit. The developed ALT kit based on *ld*LDH was found to accurately detect ALT activity in serum, with a margin error of <5% compared with the commercial kit. In addition, its sensitivity and detection range met the kit requirements for clinical applications. This study lays the foundation for the development of new ALT kits.

## 5. Conclusion

*Ld*LDH is the key enzyme in ALT kits and has important application value in clinical detection of disease. In this study, *ld*LDH from *L. delbrueckii* was expressed in an *E. coli* expression system, purified, and a series of studies were conducted. The results showed that *ld*LDH had high specific activity and thermal stability, with a specific activity of 1,864 U/mg. When placed at 50°C for 20 min, enzyme activity was not lost, and the kit met the requirements for clinical diagnosis. The use of sucrose as a stabilizer significantly improved *ld*LDH stability during freeze-drying and storage. The results of the ALT test kit prepared with *ld*LDH were consistent with those reported using commercial kits. Based on the findings of this study, the developed ALT kit meets the requirements for clinical diagnostic application.
